# New approach for T-wave end detection on electrocardiogram: Performance in noisy conditions

**DOI:** 10.1186/1475-925X-10-77

**Published:** 2011-09-09

**Authors:** Carlos R Vázquez-Seisdedos, João Evangelista Neto, Enrique J Marañón Reyes, Aldebaro Klautau, Roberto C Limão de Oliveira

**Affiliations:** 1 Centre for Studies in Neurosciences and Images and Signals Processing (CENPIS), Electrical Engineering Faculty, University of Oriente, Santiago de Cuba, Cuba; 2 Electrical Engineering Faculty/Federal University of Pará, Belém, Brazil; 3 Paulista University, Manaus, Brazil; 4 Electrical Engineering Faculty/State University of Amazonas, Manaus, Brazil

## Abstract

**Background:**

The detection of T-wave end points on electrocardiogram (ECG) is a basic procedure for ECG processing and analysis. Several methods have been proposed and tested, featuring high accuracy and percentages of correct detection. Nevertheless, their performance in noisy conditions remains an open problem.

**Methods:**

A new approach and algorithm for T-wave end location based on the computation of Trapezium's areas is proposed and validated (in terms of accuracy and repeatability), using signals from the Physionet QT Database. The performance of the proposed algorithm in noisy conditions has been tested and compared with one of the most used approaches for estimating the T-wave end point: the method based on the threshold on the first derivative.

**Results:**

The results indicated that the proposed approach based on Trapezium's areas outperformed the baseline method with respect to accuracy and repeatability. Also, the proposed method is more robust to wideband noise.

**Conclusions:**

The trapezium-based approach has a good performance in noisy conditions and does not rely on any empirical threshold. It is very adequate for use in scenarios where the levels of broadband noise are significant.

## Background

The Electrocardiogram (ECG) analysis is the heart diagnostic technique most used in the clinical practice due to its excellent benefit-cost relationship. From the ECG signal, the following features are evaluated: amplitude, morphology and duration of its waves, intervals and segments as well as their appearance sequence.

**Figure 1:**
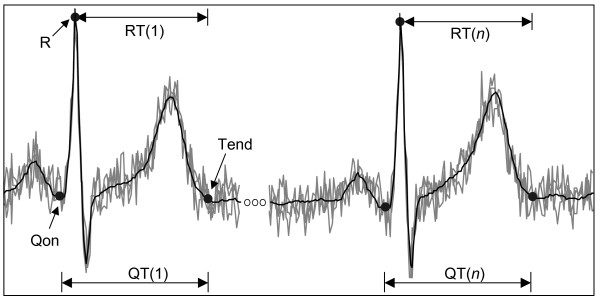
**The beat-to-beat analysis of the QT (or RT) interval variability in presence of wideband noise requires an accurate T-wave end detection because of the small variability of the QT (or RT) interval**.

The diagnostic using the ECG signal has numerous approaches. One of them is the beat-to-beat analysis of the time intervals between the Q-wave onset and the T-wave end or interval QT (see Figure 1) during periods of time, typically, from 5 min to 24 hours. Sometimes the interval QT is estimated as the time interval between the peak of the R-wave and the end of the T-wave (RTe). The QT or RT intervals depend on the accuracy with which both points (onset and offset) are determined, especially of the T-wave end (Tend) due to the slow transition in the signal around this point, eventually contaminated by noise and interference on ECG signal.

Three variants of studies with QT interval have been done: (a) QT mean duration or QT length (QTL), (b) QT time variability or QT variability (QTV), and (c) spatial variability or QT dispersion (QTD). The last two ones are measures of the ventricular repolarization (VR) heterogeneity degree and they are typically computed using the standard deviations of QT intervals series measured through the time or the different ECG leads.

The prolongation of QTL was reported as a predictor of sudden death in patients with myocardial infarction [1]. QTD and QTV are techniques relatively recent in comparison with QTL, but there are reports of studies showing high QTV for isquemic patients [2] and high QTD as a marker of tachycardia ventricular [3].

As mentioned before, ECG signals are very often contaminated by noise and interferences. In [4] several sources of these are described and modelled: electromiographic (EMG) noise (due to muscle contractions), instrumentation noise generated by electronic devices, electrode contact noise, motion artefacts, electrosurgical noise, power-line interference and base-line drift due to respiration. In situations of high physical activity (ex. during the realization of physical exercises or stress tests), the EMG noise is the main source of error in the Tend detection because their random nature strongly affects the slow transition speed around each T-wave end. This type of noise has broad-band frequency characteristics which overlap with the frequency spectrum of T-wave, and also occur in instrumentation noise. The motivation of this work has been to research an algorithm for Tend detection that is the least sensitive to the presence of broad-band noise or Gaussian white noise (WN). The detection of T-wave end (Te) point on ECG with high accuracy is determinant for QTV analysis because of its small variability (few milliseconds), mainly, in presence of broadband noise. For instance, if a sampling period is equal to 4 ms, a detection error of 4 samples (16 ms) could introduce a negative bias on the diagnostic.

Various methods have been proposed for detection of Tend point based on: intersection of lines [5], threshold on the amplitude of T wave [6], threshold on the first derivative of ECG signal [7], computation of: distances [8], angles [9] and areas [10], correlation with a template [11], mathematical models of ECG [12], and wavelet transform [13], among others methods. All have some advantages and some drawbacks in relation to complexity, computational cost, waveforms morphological variations, noise sensitivity and Tend dependence on threshold. It is not the purpose of this paper to review all the existing methods for Tend point detection. Instead, we will briefly summarize one of the methods mentioned earlier, because of its popularity over the years and in order to contrast the novelty of the method proposed in this paper.

### I. Tend point detection based on threshold method on the first derivative (THD)

This method is based on the principle that the derivative of an isoelectrical segment (after reducing the noise and eliminating the line base drifts) is approximately null, while the derivative of the ECG waves, is not. It defines the T-wave end as the point where the derivative crosses a certain threshold proportional to the T-wave derivative maximum absolute value [7].

**Figure 2:**
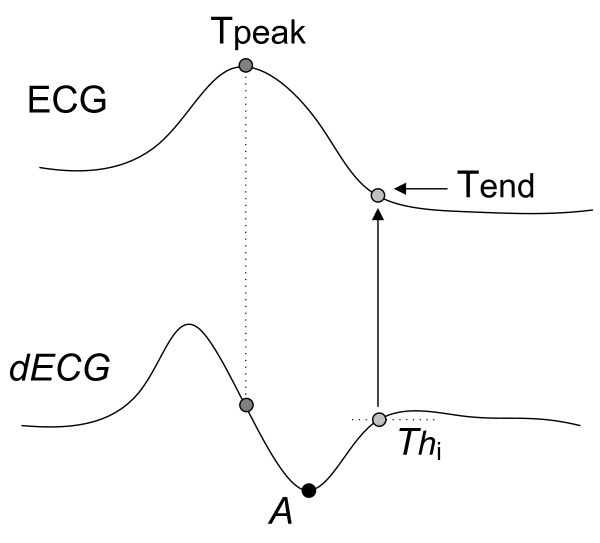
**Threshold method on the first derivative applied to a positive T-wave**. A threshold for the *i*-beat (*Th*_i_) is calculated by dividing the minimum derivative value by an empirical factor (*K*). The first sample below *Th*_i _is defined as the T-wave end.

Figure 2 shows the implementation of this method for a positive T-wave morphology. The upper half signal corresponds to the T-wave of the ECG signal, and the lower half signal is its derivative (*dECG*). For this morphology, first, the point of minimum derivative is computed (A = min (*dECG*)), second, this value is divided by a constant *K *(proportionality factor), obtaining a threshold value equal to *Th*_i _(= A/*K*). The T-wave end point is defined as the first forward sample where the value of the first derivative of the T-wave downslope became smaller than a threshold value *Th*_i _transferred to the original signal.

When *K *is high (for example, 10), the detected point will be nearer to the isoeletric segment, otherwise it will be nearer to the point of minimum (maximum) slope after the T-wave peak for a positive (negative) T-wave. This method has been applied to several studies, and demonstrated as robust. It is cheaper in terms of numerical computation and very useful to determine the T-wave end for signals with small T-P segments (for example, during intense exercise) because it predicts the Tend point from the computation of the maximum (or minimum) slope of the last segment of T-wave and doesn't need any reference point in the TP segment. However, it has the problem of the empiric selection of the threshold that must be adapted to the level of an eventually non-stationary noise.

Up to our knowledge there are only three studies about the influence of noise on the accuracy of the T-wave end detection. In [14] the R-Tend interval (from the R-peak to the T-wave end) is analysed using two computer-generated ECG signals with a single morphology of the T-wave. In [15], the effect of noise is analysed based on the statistical indexes computed, again, from eight different estimations of "QT interval" time series, using simulated signals with only 6 different morphologies. In both papers the results express the measure of a differential interval that depends on the onset and offset positions simultaneously and not only on T-wave end, as in this work. In [16] the Tend location error is studied by adding random noise to fifty morphologies of synthetic ECG recordings, but not using real signals.

The aim of this paper is to propose a new approach for the location of T-wave end, and show its high performance (in terms of accuracy) in presence of noise using several morphologies of real signals from QT Database (QTDB) [17]. The proposed method is compared to the previous method, which was chose due to its wide use.

## Methods

It is convenient to clarify that this paper considers only T-wave end detection. Obviously, the R-wave point needs to be estimated first in order to delimit an interval that contains the T-wave. Because the R point detection has been broadly described, no further discussion on this subject is pursued in this paper. Any R-wave detector with demonstrated robustness can be used. In [18] there is an extensive review of recent approaches for R-wave detection.

**Figure 3:**
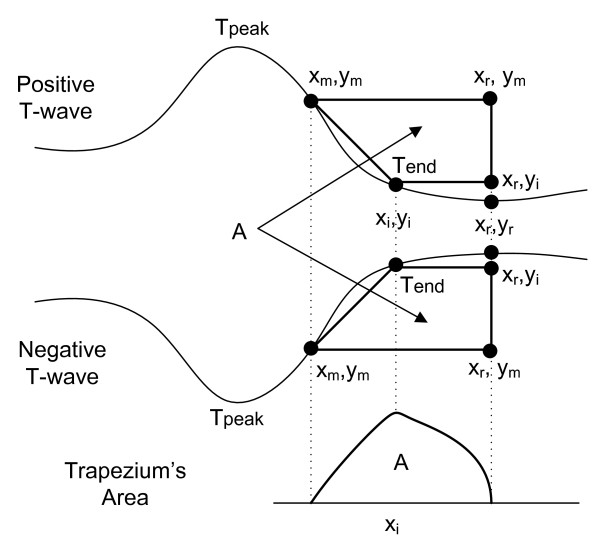
**Determination of the T-wave end (for a monophasic wave) by the computation of the areas of several trapezes formed by three fixed points and one mobile (x_i_, y_i_)**. The Tend corresponds to the point where the area A is maximum.

Let us first consider monophasic T-waves (positive or negative) as shown in Figure 3. Any other morphologies, once identified, can be treated as a particular case of this one, as will be described in the following section.

### I. Method and Algorithm of the Trapezium's Areas

The trapezium's area (TRA) approach presupposes that T peaks are located, through the search of maxima and local minima in a window whose beginning is the previous peak of the R wave. During this search, the morphology can be also identified using some existing approaches, for example, the method proposed on [7]. Since our objective is to characterize the accuracy of the new T-end detector in presence of noise, we will consider an ideal detector which provides several values for the T-wave peaks positions, similar to those annotated by the cardiologists.

The TRA method is based on the calculation of successive areas of a rectangular trapezium with three fixed vertexes and one mobile vertex: (*x_i_, y_i_*), which is shifted through the signal, from (*x_m_, y_m_*) to (*x_r_, y_i_*), while the total area is computed. T-wave end is defined as the point where the area A of the trapezium is maximum (Figure 3).

The formula of the area of the trapeze is:

(1) $$A=0.5\left(y_{m}-y_{i}\right)\left(2x_{r}-x_{i}-x_{m}\right)$$

where:

• (*x_m_, y_m_*) is the abscissa and the ordinate, respectively, of a point with the highest absolute derivative inside the T-wave and after the last peak (maximum or minimum). The derivative value on this point is a minimum negative for positive T-waves and is a maximum positive for the negative T-waves.

• (*x_r_, y_r_*) is the abscissa and the ordinate, respectively, of a reference point located on the T-P isoelectric segment. The exact location is not very important as long as the point is beyond the T-wave end.

• (*x_i_, y_i_*) is the abscissa and ordinate, respectively, of a mobile point among the two points mentioned before.

As shown in Figure 3, the area A will be a:

• minimum or zero when (*x_i_, y_i_*) is on the vertexes (*x_r_, y_r_*) or (*x_m_, y_m_*), respectively.

• maximum when (*x_i_, y_i_*) is on the end of the T-wave

The TRA algorithm is based on the method described previously. The steps of this algorithm are the following:

#### Pre-processing

1) High-pass filtering of the ECG signal (Butterworth, zero-phase, 4^th ^order, cut-off frequency equal to 0.5 Hz) to reduce baseline wander.

2) Low-pass filtering of ECG obtained in (1) (Butterworth, zero phase, 4^th ^order, cut-off frequency equal to 30 Hz) to reduce noise.

#### Processing (assuming T-wave peak positions)

3) Determination of the point identified as "*x_m_*" located in the segment after the T peak, which has a minimum (maximum) value in the first derivative, and be after the maximum (minimum) for a T-wave positive (negative). For that, the algorithm searches in a 200 ms window, starting from the T-wave peak (maximum or minimum). This segment is appropriate to embrace the *x_m _*point in the final segment of the wave T.

4) Determination of a point identifies as "*x_r_*" located inside the isoelectric segment and searched in a window between 200 ms and 400 ms, from the peak (maximum or minimum) of the T-wave, preferably with a value of the first derivative near to zero. If no point satisfies this condition, the central point is chosen. Actually, the exact position of this point is not very important as long as it is beyond the T end point.

5) Calculation of the trapeziums areas of all the points located between "*x_m_*" and "*x_r_*".

#### Decision rule

6) Identification of the point with maximum area identified as the T-wave end.

**Figure 4:**
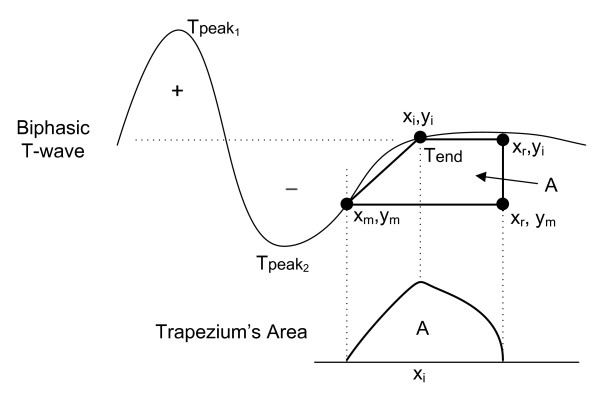
**Determination of the T-wave end for biphasic (positive-negative) morphology**.

As shown in [13], T-wave morphologies can be classified as positive, negative, biphasic (positive-negative or negative-positive), ascending-only, and descending-only. So far, the TRA algorithm has only been explained for monophasic T waves (positive or negative). For the case of two-phase waves (positive-negative or negative-positive) or the only upwards or downwards, the point (*x_m_*,*y_m_*) should be chosen in such a way that the wave section between this point and the (*x_r_,y_r_*) point has a monophasic behaviour, either rising or falling, as shown in Figure 4 for a positive-negative morphology.

### II. Evaluation of the Trapezium's Areas Method: database and parameters

The evaluation of the Trapezium's areas method was performed with the QTDB, which constitutes a standard to validate and compare the T-wave end detection algorithms.

This database consists of 105 15-min two-lead ECG recordings sampled at 250 Hz. It includes a variety of T-wave morphologies chosen from several MIT-BIH databases (Arrhythmia, Supraventricular Arrhythmia, Normal Sinus Rhythm, ST Change Database, Long-Term Database, Sudden Death) and European ST-T Database. In 105 records, 3542 T-wave ends have been annotated by one cardiologist and in 11 of these records another cardiologist annotated 402 T-wave ends, being a total of 3944 beats. In each record, at least 30 beats have been manually annotated by cardiologists, labeling the end of the T wave (and others fiducial points). We discard some beats of poor quality for T-wave end location: 703 of the 3542 annotated by cardiologist 1 and 129 of the 402 annotated by cardiologist 2, to give a total of 3112 detected beats.

Traditionally [18], four parameters have been used in the detector's validation:

Sensitivity, Positive Predictivity, the mean and the standard deviation of the detection errors, that is, the time difference between the automatic and the cardiologist annotation. In this work, we only computed the last two ones because of our assumption that the R and T peaks are located by an exemplar method (S = 100%, P = 100%).

Two evaluation criteria are implemented to compute the detection errors:

1. Best beat per record (BB): the best result that minimizes the detection error among the two T-wave end computed positions is chosen as the real Tend. This procedure was first adopted in [13] and later in [10]. The justification given in [13] is that the cardiologist has made his annotation by looking at both leads and his decision is based on the best lead. In clinical practice, this criterion requires a robust automatic decision rule.

2. Best lead per record (BL): the best ECG lead which contributes with the biggest number of T-wave end points, according to the previous criterion, is chosen [19]. If the contributions of Tend are equal, the first lead is selected. This procedure is more realistic from the viewpoint of a human operator.

The procedure to compute the global mean (*me*) and standard deviation (*sd*) of the detection errors for each evaluation criterion has the following steps:

(1) for each annotated beat (by both cardiologists) on each record, the detection errors are computed,

(2) for each record *i*, the mean (*M*_i_) and standard deviation (*S*_i_) of the detection errors are calculated,

(3) for all records, the mean of all *M*_i _(*me*) and the mean of all *S*_i _(*sd*) are computed.

### I. Performance in noisy conditions: Comparison between methods

Since each original approach (and its corresponding algorithm) uses different types of filtering, their performance could depend on the characteristics of the filters. To homogenize this dependence, the pre-processing used for the algorithm of the trapezes was the same for the first derivative method.

To be consistent with the clinical practice, for each record of QTDB, the "best ECG lead" was selected. As the signal-noise ratio (S/N) for each T-wave (in the same lead) is different, it is not feasible to add noise by controlling the S/N ratio of the global lead. To guarantee a uniform noise level the control parameter will be the T-wave peak amplitude (*A*_TWP_) beat by beat. Broadband noise was simulated as zero mean WN added to ECG signal.

The procedure to characterize the performance in the presence of noise by each method consists of the following steps:

1. High-pass filtering followed by low-pass filtering like the pre-processing described for the TRA algorithm.

2. Obtain the reference T-wave end using the method *X *(*T_RX_*). The sub index "*X*" will be "D" for the threshold on the first derivative method; and T for the trapezium's areas method.

3. Compute the reference T-wave peak amplitude (*A*_TWP_) using the values of expert's annotations.

4. For each beat of filtered ECG signal, add WN of amplitude equal to *N% of A*_TWP_, *N *= {1%, 5%, 10%, 20%}. For each level of noise, WN was generated 200 times and added back to filtered ECG signal; then it was low-pass filtered like in step 2, and the mean of 200 respective estimates of T-wave ends was computed. This value becomes the T-wave end for the level of noise *N *and method *X *(*T_NX_*).

5. Obtain the successive estimates of the T-wave end for each beat *i*, level of noise *N *and method *X *(*T_NXi_*).

6. Compute the modular percentage relative error (*E_NX_*) for the algorithm "*X*" and level of noise *N *according to the following expression:

(2) $$E_{NX}=\bar{\overset{k}{\underset{i=1}{\sum }}\left|\left(\frac{T_{NXi}-T_{RXi}}{T_{RXi}}\right)\right|}\times 100$$

where,

*k *is the total number of beats annotated by both cardiologist (3112)

*T_NXi _*is the *i-th *T-wave end obtained by algorithm *X *when the level of noise is *N% of A*_TWP_. *N *= {3%, 5%, 10%, 20%},

*T_RXi _*is the *i-th *reference T-wave end for the algorithm *X*. For the algorithm of threshold on first derivative (THD), we use the following threshold factors: *K *= 2 (50%), *K *= 5 (20%) and *K *= 10 (10%).

*E_NX _*is the overall mean of the modular relative-detection-errors due to added noise. It gives an idea of the upward or backward displacement (i.e. absolute) of the Tend position due to the effect of the noise. Therefore, *E*_NX _is a measure of the method performance in noisy conditions. Signal processing was done on Matlab 7.7 (The MathWorks, Inc, Natick, MA).

## Results and Discussion

### I. Evaluation of the Trapezium's algorithm

Table 1 shows the results of validation of the proposal algorithm for the two evaluation criteria. The *me *value expresses how close the detector is to the annotated markers (accuracy), and *sd *value provides information about the stability (repeatability) of the detection criteria. The numerical results of the threshold algorithm were chosen from [13] and [19] for the criteria of the best simultaneous beat per record and the best lead per record, respectively. The results for the THD algorithm is for the case of a threshold factor equal to 2, which was reported in [7] as the threshold with the best performance.

**Table 1:** Mean (*me*) and standard deviation (*sd*) of the differences between the automatic and the cardiologist annotation for both methods and both evaluation criteria.

Best beat per record (BB)			Best lead per record (BL)	
	**TRA**	**THD **[12]	**TRA**	**THD **[18]

*me*	-2.29	13.5	-1.98	18.68
*sd*	7.15	27.0	16.46	29.79

TRA: Trapezium's Areas, THD: threshold on the first derivative. The time unit is millisecond.

The results of Table 1 show that, in terms of error mean value and SD, the proposed algorithm outperforms the other compared algorithm for both criteria. By examining the errors of the proposed algorithm, it has been observed that the large errors are mainly due to the incorrect elimination of the line base drifts when it changes abruptly. Thus, it is important to develop more accurate and robust methods (ex. adaptative or non linear filtering methods) for eliminating the line base drifts without deforming the morphology of the T-wave final segment.

The obtained results are very similar to those reported by other T-wave end detectors [10-13,16], because the slight differences (in the mean detection error) are smaller than or around one sample (4 ms). The standard deviation for our algorithm is around two (three) samples for the BB (BL) criterion, which is within the expert tolerance limits (30,6 ms for the standard deviation) [20] and presents an excellent repeatability value in comparison with several algorithms presented in the last ten years [10-13,16]. As we know, the annotation of the T-wave end has not been adopted yet by specialists causing a high standard deviation among specialists.

### II. Comparison between algorithms: Performance in noisy conditions

**Figure 5:**
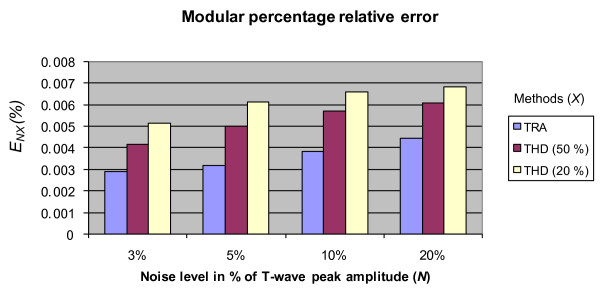
**Modular percentage relative error versus noise level for both algorithms: TRA and THD**. The value of the several hypothesis tests (T-student) for each algorithm with regard to the algorithm of the trapezes was upper bounded by a probability *p *≤ 8.9 × 10^-7^.

Figure 5 shows the values of the modular percentage relative error (*E*_NX_) for each noise level and algorithm. In all cases, the error with the TRA algorithm is smaller than the one obtained with the THD algorithm with a high degree of significance (*p *≤ 8.9 × 10^-7 ^or even smaller). This result shows the better best performance of TRA in the presence of low, middle and high levels of noise.

Figure 5 does not include the results for *K *= 10 (10%) because, in this case, the errors are extremely high and the significance level hypothesis test are low or not of statistical significant, as shown in Table 2.

**Table 2:** Comparison of modular percentage relative errors (*E*_NX_) between the Trapezium's algorithm and THD algorithm (threshold factor is equal to 10%).

*E*_NX_	3%	5%	10%	20%
TRA	0.0029	0.0031	0.0038	0.0044
THD 10%	0.049	0.050	0.093	0.137
Significance level	*p *≤ 0.071	*p *≤ 0.135	*p *≤ 0.047	*p *≤ 0.047

For the threshold of 50%, the mean error of the THD algorithm is the smallest, reaffirming the results described in [7] and is highest for the threshold *K *= 10. In [7], it was only considered the case with *K *= 2 (50%) because experimentally it showed the best performance. Nevertheless for *K *= 2, the T-end point is more far from the true end.

The better performance in the presence of noise of the TRA algorithm can be justified because the computation of areas corresponds to an integration process and therefore, attenuates the noise (low-pass filtering effect). In contraposition, the differentiation process that is implicit in the threshold algorithm is equivalent to a high-pass filtering and therefore, amplifies the effects of noise (high pass filtering effect).

**Figure 6:**
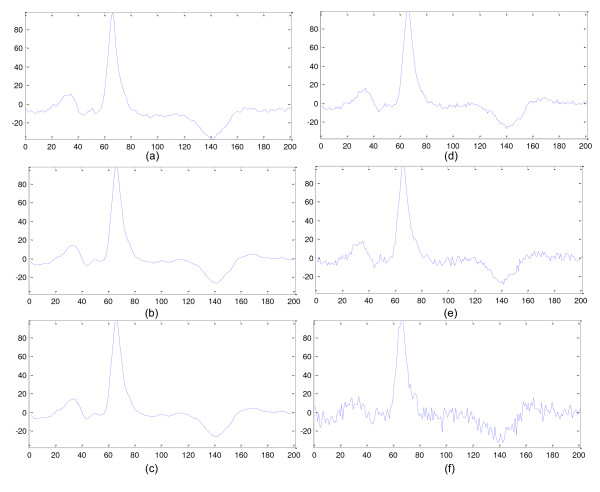
**Example of a beat of QTDB (from sel32 recording, first channel) with different levels of noise: (a) Original beat, (b) Band-pass filtered version of (a), (c) 3 of noise of *A*_TWP_, (d) 5% of noise of *A*_TWP_, (e) 10% of noise of *A*_TWP _and (f) 20% of noise of *A*_TWP_**.

Figure 6 shows an example of an ECG beat (from record "sel32" of QTDB) with different noise levels. The description of Figure 4 is explained in the caption of the figure. The band-pass filtering is the combination of high-pass filtering (step 1) and low-pass filtering (step 2) of the procedure described before.

The selected noise levels are very high in some cases, mainly for some records of the QTDB which have high levels of noise where (and in spite of the low-pass filtering) the added levels increase considerably the present noise. For other practical situations, the level of noise is smaller, and the most attractive (distinctive) feature of the proposed algorithm is that it doesn't use any threshold factor, independently of the operation conditions (noises, interferences and devices).

## Conclusions

This work presented the algorithm of the trapezes, a new method to estimate the T-wave end that presents a low computational cost and mathematical simplicity. The proposed method showed a good performance in noisy conditions and it does not depend on any empiric threshold factor. The obtained results suggest the adoption of the Trapezium's approach in scenarios where the ECG is strongly contaminated by noise. The use of this approach could be extended to delineate the onset and offset of the other waves in ECG.

## Competing interests

The authors declare that they have no competing interests.

## Authors' contributions

CRVS and JEN contributed equally in the: conception and design of the study, proposal of the method, implementation of the algorithm, testing and analysis of the software simulations, realization of the statistical analysis and writing of the manuscript. EJMR, AK and RCLO helped to analyze and to interpret the results, and critically reviewed the manuscript. CRVS and RCLO participated in the coordination of the paper. All authors read and approved the final manuscript.
